# How target-orientated is the use of homeopathy in dairy farming?—A survey in France, Germany and Spain

**DOI:** 10.1186/s13028-019-0463-3

**Published:** 2019-07-12

**Authors:** Diana Keller, Isabel Blanco-Penedo, Manon De Joybert, Albert Sundrum

**Affiliations:** 1 Department of Animal Health and Animal Nutrition, University of Kassel, 37213 Witzenhausen, Germany; 2 Department of Animal Welfare, IRTA, 17121 Monells, Spain; 3 Department of Clinical Sciences, SLU, 75007 Uppsala, Sweden; 4 BIOEPAR, INRA, Oniris, 44307 Nantes, France

**Keywords:** Dairy cattle, *lege artis* treatment, Monitoring treatment outcome, Use of homeopathy

## Abstract

**Background:**

Veterinary remedies are intended to support animals in their recovery from diseases. Treatment outcome depends not only on the general effectiveness of the remedies themselves, but also on other prerequisites. This is true for antibiotics, but even more so for treatments with homeopathic products which are characterised by their individualised approach. While the effectiveness of homeopathy has been addressed in various clinical control trials, the practical conditions under which homeopathic products are used on dairy farms have not yet been investigated. This study provides an initial insight into the existing prerequisites on dairy farms for the use of homeopathy (i.e. the consideration of homeopathic principles) and on homeopathic treatment procedures (including anamnesis, clinical examination, diagnosis, selection of a remedy, follow-up checks, and documentation) on 64 dairy farms in France, Germany and Spain. The use of homeopathy was assessed via a standardised questionnaire during face-to-face interviews.

**Results:**

The study revealed that homeopathic treatment procedures were applied very heterogeneously and differed considerably between farms and countries. Farmers also use human products without veterinary prescription as well as other prohibited substances.

**Conclusions:**

The subjective treatment approach using the farmers’ own criteria, together with their neglecting to check the outcome of the treatment and the lack of appropriate documentation is presumed to substantially reduce the potential for a successful recovery of the animals from diseases. There is, thus, a need to verify the effectiveness of homeopathic treatments in farm practices based on a *lege artis* treatment procedure and homeopathic principles which can be achieved by the regular monitoring of treatment outcomes and the prevailing rate of the disease at herd level. Furthermore, there is a potential risk to food safety due to the use of non-veterinary drugs without veterinary prescription and the use of other prohibited substances.

## Background

The use of homeopathic products has experienced a popular revival in recent years. The reasons for this increased use are manifold and include the high current consumption of antimicrobial products in food-producing animals in Europe, increasing pathogen resistance to antibiotics [1] and expectations consumers have towards foodstuffs without antimicrobial residues. Very low or no withdrawal periods might also contribute to an increased use of homeopathic products in food-producing animals [2]. EU regulations on organic agriculture even promote the use of homeopathy: “homeopathic products shall be used in preference to chemically-synthesized veterinary products provided that their therapeutic effect is effective for the species of animal, and the condition for which the treatment is intended” [3]. Homeopathy, as an individualised treatment method, is quite challenging, particularly for lay people. One basic principle of homeopathy, “*similia similibus curentur*”, is to find the remedy that best matches all symptoms and characteristics in the diseased animal. People administering treatment have to select the most appropriate remedy from thousands of different homeopathic medicinal products available on the commercial market [4]. The selection of an appropriate remedy therefore requires expertise and experience in homeopathy and being familiar with homeopathic principles. Moreover, a medical treatment comprises several steps as part of a *lege artis* treatment procedure which includes an anamnesis and clinical examination, formulation of a diagnosis, selection of an appropriate remedy and evaluation of the therapeutic outcome [5]. Documentation also plays a key role in a target-orientated treatment process as it can establish the effectiveness of the process and identify changes in animal health or other clinical symptoms. While the effectiveness of homeopathy has been addressed in various studies [6–8], the practical conditions under which homeopathic treatments are being used on farms have not yet been investigated. The objective of this study was to assess the extent to which farmers consider homeopathic principles and implement a *lege artis* treatment concept in cases of mastitis which is, according to Leon et al. [2] and Roderick and Hovi [9], often treated homeopathically in dairy farming.

## Methods

### Study design

The study was conducted on 49 organic and 15 conventional dairy farms in France (organic n = 20), Germany (conventional n = 5, organic n = 15) and Spain (conventional n = 10, organic n = 14) from January until April 2015. Farmers that were identified as frequent users of homeopathic remedies in a preceding study [10] were invited to participate in the current study. In addition, an internet search [keywords: (organic) dairy farming and use of homeopathy] was performed and veterinary practitioners were contacted followed by a telephone call to the farmers found in order to achieve the required sample size of a minimum of 20 participants per country. The survey was based on a questionnaire with a total number of 25 questions designed specifically to identify the prerequisites when treating an animal using homeopathy. Open- as well as closed-ended questions were used. The questionnaire was developed by scientists (DK, IBP and MDJ) and veterinarians experienced in homeopathy from the International Association for Veterinary Homeopathy (IAVH) and was then translated into the respective national languages (DK, IBP and MDJ). The development phase was followed by an on-farm test phase where the questionnaire was employed and revised. The first part of the questionnaire (the researcher’s responsibility) focused on general farm management including animal observation practices, diagnostic procedures where disease was suspected, inspection of the stall pharmacy and measures for the early detection of diseases. Questions dealing with the identification of a *lege artis* homeopathic treatment procedure (performance of anamnesis, diagnosis, selection and application of homeopathic remedies, follow-up checks and documentation) implemented by farmers were covered in the second part of the questionnaire and conducted by the veterinarian from IAVH. The questionnaire also addressed the farmers’ knowledge of homeopathic principles, their homeopathic education and their attitude towards seeking veterinary advice. All homeopathic questions were based on the principles of individualised homeopathy. Farmers were interviewed according to a standardised procedure, beginning with the inspection of the stall pharmacy followed by a face-to-face interview with the farmer. Farm visits lasted from approximately 120 to 240 min. All of the respondents’ answers were recorded using an online survey tool (LimeSurvey software package©). After completing the data recording, one Excel file was extracted. The farmers’ responses to each question addressing certain prerequisites (e.g. anamnesis procedure, selection of remedies, documentation) were subsequently evaluated by one of the researchers (DK).

### Content of the questionnaire

A fundamental basic education in homeopathy plays a key role and is expected to have a strong effect on the homeopathic treatment procedure. Farmers were therefore asked what kind of basic training courses they had participated in, how many further training courses (ongoing education) they had attended in the last 3 years and how long they had been using homeopathy. Although multiple answers to this question were allowed, only the most extensive training course was selected for the evaluation (for example, where “part time, i.e. evening or weekends, totalling 1–2 days” and “full time totalling 1 week to 1 month” were the given answers, only the latter answer was considered in the evaluation).

A thorough anamnesis is essential in formulating a diagnosis which influences the appropriateness of the homeopathic treatment and the corresponding choice of remedy. The process of anamnesis involves, *inter alia*, recalling the most relevant sections of the animal’s history. Respondents were thus asked where they obtained the historical health records of the diseased animals (multiple answers were permitted). Homeopathy requires careful observation of an animal in order to detect early on the smallest changes in animal health and behaviour, as early treatments may offer the best prospects for success. Thus, farmers were asked how much time per day they spent observing their animals at herd level (results are based on the farmers’ self-assessment). Studying the unique signs and symptoms of the diseased animal characterises a homeopathic clinical examination. The more striking, uncommon and peculiar the symptoms found, the higher the chance of selecting the most suitable homeopathic remedy [11]. Both general and undefined symptoms (such as loss of appetite and fever) require little attention as they are observed in almost every disease and prompt the use of almost every remedy. Formulating a diagnosis is the process of identifying the nature of an illness and relies on thorough anamnesis and clinical examinations. This process is often challenging for lay people. Hence, the respondents were asked how often they sought the expertise of a homeopathic professional/veterinarian in treatment decisions. Farmers were also asked to illustrate whether, and if so how, they performed a comprehensive clinical examination and what kind of diagnostic measures they used. It is crucial to identify the type of bacteria present in the udder prior to starting any kind of mastitis treatment. Where homeopathic treatment is unsuccessful, the results of the laboratory milk analysis can be used for conventional mastitis treatment. The farmers were therefore asked for their diagnostic procedure before they started a mastitis treatment.

**Fig. 1:**
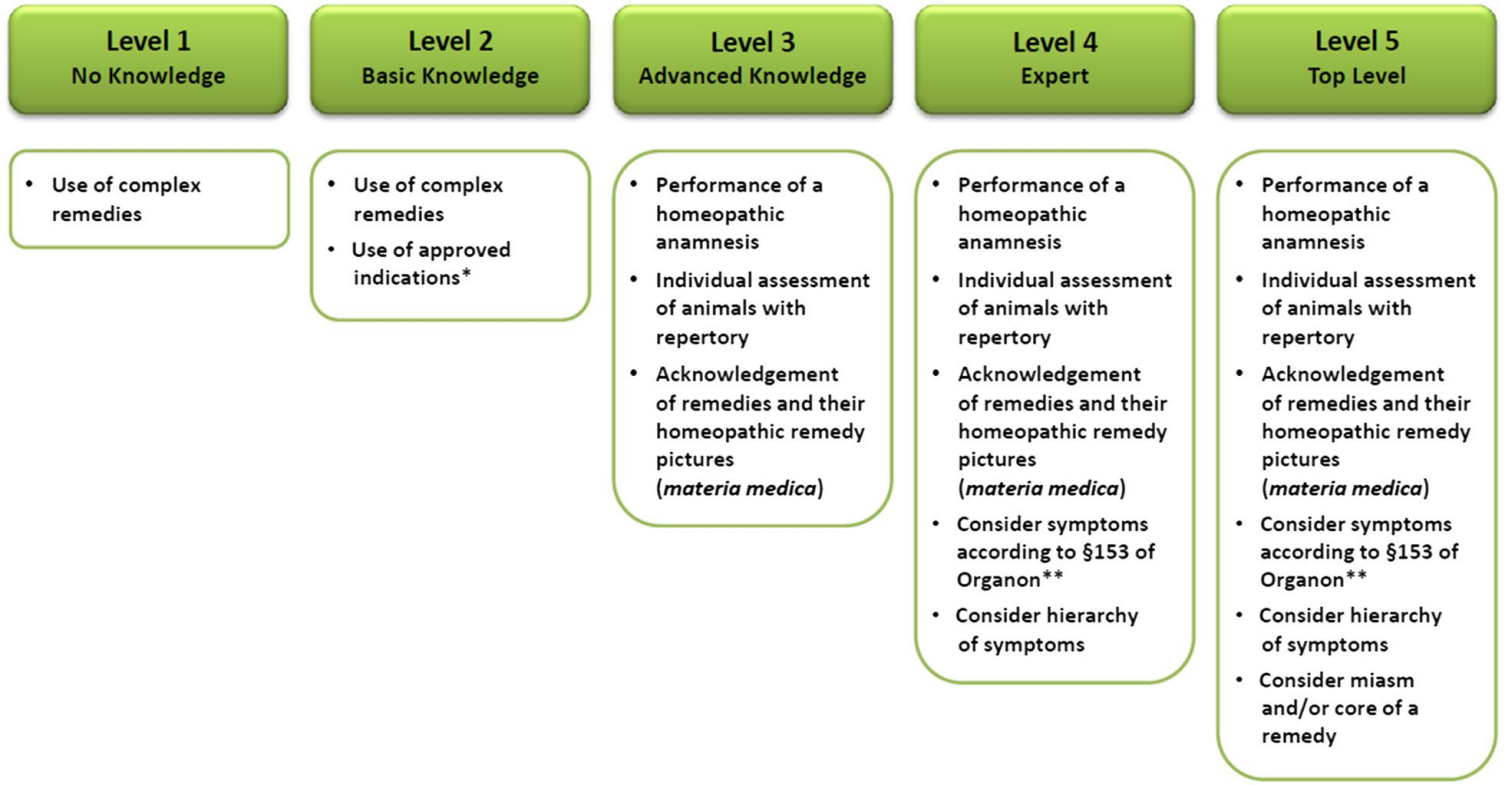
Levels of awareness of the homeopathic principles. Single asterisk—simplified selection of a homeopathic remedy on the basis of clinical diagnosis and limited leading symptoms. Double asterisk—§153 of Organon of medicine: the more striking, singular, uncommon and peculiar the symptoms found, the higher the chance of selecting the most suitable remedy. General and indefinite symptoms (loss of appetite, debility and fever etc.) require little attention if they cannot be more accurately described

Hahnemann [11] hypothesised the principle of “*similia similibus curentur*”, stating that the characteristics of the diseased animal must be similar to the characteristics of the “remedy picture”. A remedy picture is a collection of physiological and psychological symptoms caused by a particular homeopathic remedy in a healthy animal. Homeopathic practitioners use usually repertories containing symptom pictures (a list of signs and symptoms and the corresponding homeopathic remedies that they are thought to be effective for) and Materia Medica containing remedy pictures (a record of different homeopathic remedies and their description of the clinical picture which they cause for selecting an appropriate homeopathic remedy) [12, 13]. In order to achieve the best selection, a repertorisation (a cross-check of the clinical symptom picture with the corresponding remedy picture) is necessary. Selecting the correct remedy requires expertise and experience in homeopathy and various homeopathic principles need to be considered during the selection process. Farmers were thus asked which reference sources they used for choosing homeopathic remedies, due to the challenges in selecting an appropriate homeopathic remedy. The farmers’ level of awareness of homeopathic principles was assessed by the veterinary experts in homeopathy and categorised using predefined levels (Fig. 1). A further principle of individualised homeopathy is the prescription of only one remedy at a time as the prescriber cannot distinguish which component of a complex remedy was effective and predicting the interactions which might occur between given remedies is not possible. Therefore, farmers were asked what percentage of homeopathic single remedies and complex remedies they used for treating mastitis. Checking the outcome of the treatment administered is also important when using remedies, being, amongst other things, responsible for a delay in the change to other more effective medical treatments, independent of the particular treatment method. Delaying treatment which would otherwise have been more effective has a lower prospect of success, since valuable time has elapsed. In this study, the respondents were asked how they check treatment outcomes. Finally, documenting treatments is important for various reasons. Firstly, people who treat food-producing animals are required by EU and national legislation to document every treatment given to diseased animals [14, 15]. This compulsory documentation serves to ensure the protection of public health. Secondly, there is always the risk that treatment is not successful and that the therapy or remedy has to be modified. The initial symptoms might have changed due to the previous treatment. Without documenting the initial symptoms, it is difficult—if not impossible—to find an appropriate remedy. Using the documentation, the prescriber is able to review the previous treatment process and alter or optimize the treatment strategy immediately. More importantly, documentation will help the prescriber to ascertain whether the treatment given was successful or not. For these reasons, the questionnaire also dealt with the farmers’ documentation procedures. In order to evaluate how comprehensively farmers documented, they were asked to choose from one of three possible options: never, partially or every time (meaning that all treatment steps were documented every time).

### Evaluation

For the purpose of the evaluation a frequency distribution, with or without previous categorisation of subject matter in question, was used in the current study.

For further information regarding stall construction and other farm conditions (i.e. aspects of farm management and disease prevention) relating to the current study, see the IMPRO-project report on http://www.impro-dairy.eu.

## Results

### Demographics of farms and farmers

Most of the farmers who regularly make use of homeopathic remedies were on average 45–54 years old (n = 30; 47%) and male (n = 44; 69%). Only a few of the users were female (n = 20; 31%) or were from other age groups. The results revealed that 63% of the farmers (n = 40) were members of an organic association. The estimation of farm size was based on the number of cows. The largest farms visited were in Germany (average [μ] = 118, standard deviation [s] = 215, coefficient of variation [cv] = 1.82), followed by France (μ = 61, s = 25, cv = 0.41) and Spain (μ = 34, s = 13, cv = 0.39). The average farm size of the farms visited in Germany and France was much larger than the nationwide average farm size (Germany n = 51, France n = 47). The average farm size of the surveyed farms in Spain came close to the nationwide average farm size (n = 38) [16]. Milk records were available on 91% of the farms and an evaluation of individual or detailed milk record data was performed by 85% of the farmers who kept milk records. For a detailed demographic description see Table 1.

**Table 1:** Status quo of prerequisites for the use of homeopathy present on dairy farms in France, Germany and Spain

Questions covered by questionnaire	Number of farms in		
	France	Germany	Spain
*Demographic description*			
Gender			
Female	7	7	6
Male	13	13	18
Age group			
< 26	–	1	–
26–34	3	6	1
35–44	2	3	5
45–54	10	8	12
55–64	5	2	6
Member of organic farmers association			
Yes	17	16	7
No	3	4	17
Number of cows			
Min	30	26	11
Median	58	75	30
Max	130	1000	55
Quartile Q1	44.5	49.5	27.0
Quartile Q3	75.5	81.0	46.5
Milk records available			
Yes	15	20	23
No	6	–	9
*Basic training in homeopathy*			
Duration of using homeopathy (years)			
< 1	1	–	–
1 to 5	4	2	15
5 to 10	6	6	4
More than 10	9	12	5
Basic training courses in homeopathy			
No specific training course	1	3	21
Part time: totalling 1–2 days	–	7	1
Part time: totalling > 2 days	–	1	1
Full time: 1 day–1 week	19	9	1
Full time: 1 week–1 month			
Full time: > 1 month			
Supervisor of training course			
Veterinarian	19	8	3
Professor of a university	–	–	–
Professional consultant/Advisors	–	–	–
Members of a homeopathic organisation	–	–	–
Other homeopaths/“Tierheilpraktiker”	–	9	–
*Anamnesis*			
Availability of historic health records			
No information exists	–	–	19
From memory	6	14	19
From health ledger papers/cow files	16	8	3
From herd management software	5	5	1
Duration of animal observation (min)			
1–10	12	2	2
11–20	7	6	–
21–30	1	4	2
31–40	–	–	2
> 40	–	8	18
Combined with other activities (e.g. milking routine, feeding)	19	15	19
Not combined with other activities	1	5	1
*Clinical examination*			
Type of clinical examination			
No clinical examination	2	1	–
Homeopathic clinical examination	8	9	17
Use of approved Indication (looking for leading symptoms)	6	6	–
General clinical examination (similar to allopathic treatment)	2	4	5
Help from veterinarian	2	–	2
Taking quarter milk samples			
No	13	10	11
Yes	7	10	13
In case of clinical mastitis			
For all animals	0	1	2
For selected animals	2	2	9
In case of subclinical mastitis			
For all animals	0	0	0
For selected animals	3	1	0
In case of clinical and subclinical mastitis			
For all animals	0	1	1
For selected animals	2	5	1
*Diagnosis*			
Consultation of a professional			
Never	16	7	–
In every case of illness	–	1	18
Only at selected animals	4	1	–
Only at specific diseases	–	5	6
Only if no recovery is foreseeable	–	6	–
*Availability of remedies*			
Source of homeopathic remedies^a^			
Veterinarian	7	6	15
Pharmacy	19	18	13
Internet	–	4	–
Number of homeopathic remedies stored			
Total number of different remedies	40	314	47
Minimum	3	11	0
Median	14	55	5
Maximum	20	218	24
*Selection of homeopathic remedies*			
Use of reference materials^a^			
Advice of a veterinarian	8	5	24
Internet	–	3	1
Materia medica	7	6	–
Rely on current knowledge alone	2	5	5
Repertory	7	4	–
Short manual for homeopathy	11	19	1
Software	1	1	–
Other people	4	4	1
*Treatment outcome*			
Checking treatment outcome by a veterinarian			
Yes	–	4	15
No	20	16	9
Checking treatment outcome by farmer			
Yes	20	20	13
No	–	–	11
Type of checking treatment outcome by farmer^a^			
Pure observation (visual)	20	19	20
Clinical investigation (e.g. udder palpation, CMT)	11	15	18
Laboratory investigation	–	3	–
*Documentation*			
Taking anamnestic records			
Yes	10	6	14
No	10	14	10
Taking treatment records			
No	4	12	16
Yes	16	8	8
Every time	9	–	2
Partial documentation	7	8	6

^a^More than one answer was permitted

### Basic training in homeopathy

The majority (41%) of the farmers have used homeopathy for more than 10 years, followed by the use of homeopathy from 1 to 5 years (total 33%) and from 5 to 10 years (total 25%) (Table 1). Only 1 French farmer answered that he had been using homeopathic remedies for less than 1 year. However, there was a wide variation in the quality and duration of the basic homeopathic training courses. Of the 64 farmers, 25 (39%) stated that they had not attended a specific training course and/or had taught themselves to use homeopathy using books or the Internet. Specific training courses in homeopathy were attended by 61% of farmers. All French and Spanish farmers and 47% of the German farmers who had participated in a professional course were trained by a veterinarian. The remaining 53% of the German farmers were trained in homeopathy by a non-veterinary practitioner.

### Anamnesis

A highly heterogeneous result emerged on how farmers dealt with the issue of anamnesis. A total of 79% of the Spanish farmers stated that they mostly had no historical information on the diseased animal or that they tried to reconstruct its medical history from their memory. A similar situation was found in Germany where farmers also generally obtained the medical history from memory (70%). Only 8 German farmers used information from health ledgers/cow files. In contrast, 80% of farmers in France used paper files to maintain a medical history. All in all, only 11 out of 64 farmers in the three countries made use of professional herd management software for this procedure.

A high variation was also noted in the quantity and quality of animal observations. Only 7 farmers stated that they performed an animal observation while doing nothing else. The time they took to observe their animals differed considerably and ranged from 1 to more than 40 min/day. All other farmers stated that they observed the cows in combination with other activities, for example during the milking routine, while feeding or in pasture. While French farmers observed animals for a period of 1 to 30 min, Spanish farmers took more time for this process, and claimed to often spend more than 40 min for animal observations each day. Further details are provided in Table 1.

### Clinical examination

When using homeopathy, 34 farmers (53%) agreed that a homeopathic clinical examination needs to be performed, whereas 11 farmers (17%) only looked for general clinical signs as commonly performed prior to allopathic treatment (e.g. fever or flaks in milk). In addition, 12 farmers (19%), 6 each from France and Germany, answered that they looked for typical, well-known symptoms and chose a so-called “*approved indication*” (i.e. a simplified selection of a homeopathic remedy on the basis of clinical diagnosis and limited leading symptoms). The remaining 7 farmers either did not perform a homeopathic clinical examination (5%), or they (6%) had assistance from a veterinarian during this process.

Quarter milk samples for laboratory cyto-microbiological analysis before farmers treated mastitis were never taken by 53% of farmers. The remaining 30 farmers (47%) only took quarter milk samples depending on the severity of the mastitis, effort and time for labour or course of treatment. In the case of clinical mastitis, 16 out of the 30 farmers collected milk samples (for all animals n = 13 farmers; for selected animals n = 3 farmers) while in the case of subclinical mastitis, 4 farmers collected quarter milk samples for selected animals. A laboratory milk analysis for both subclinical and clinical mastitis was performed by 10 farmers with different degrees of thoroughness. Table 1 shows a detailed breakdown of the present clinical examination procedure on farms.

### Diagnosis

The results of the evaluation show a widespread picture concerning the diagnostic procedure. While French farmers generally never consulted a professional (80%), or only in the case of selected animals (20%), 75% of Spanish farmers consulted a professional in every case of illness. The remaining 25% of Spanish farmers asked for professional advice in specific disease cases. Most German farmers either never consulted a professional (35%) or consulted a professional only in cases where no recovery was foreseeable for the diseased animals (30%) or in specific cases of disease (25%). The remaining 2 German farmers (10%) never consulted a veterinarian or only selected animals were examined by a veterinary practitioner (Table 1).

### Availability and selection of a remedy

A high variation in remedies stored on farms was found during the inspection of the stall pharmacy. In total, 324 different homeopathic remedies were identified (among them 240 pure/single remedies, 36 complex remedies and 48 nosodes [homeopathic remedies prepared from pathological material such as blood, pus, or pathogens]). While German farmers stored from a minimum of 11 up to a maximum of 218 different remedies, farmers from France and Spain stored from a minimum of 3 up to 20 and 0 up to 24 remedies, respectively (Table 1). Homeopathic remedies dedicated for human use were found on 48 farms, mainly in Germany. The majority of farmers (78%) did not consult a local veterinarian for purchasing human homeopathic remedies, purchasing them instead in pharmacies or via internet. Furthermore, colchicine and aristolochia, prohibited for use in food-producing animals, were identified on 11 farms. Purchasing homeopathic remedies from a local veterinarian was mainly made by Spanish farmers (63%), whereas this source of acquiring remedies was used by few farmers from France (35%) and Germany (30%). Additionally, 4 German farmers received their homeopathic remedies via the Internet.

French and German farmers behaved similarly in the way they used reference materials for selecting an appropriate homeopathic remedy. Both mainly used short manuals (mostly containing “approved indications”) for the selection of a remedy. As far as the principles of individualised homeopathy were concerned, only 5 farmers from France and 4 from Germany used a repertory in combination with a *materia medica* (repertorisation). In contrast, all Spanish farmers received the advice of a homeopathic veterinarian via telephone or e-mail. Using software for repertorisation of symptoms was not very popular among the farmers. The category “other” included consulting other farmers, other homeopaths or non-veterinary practitioners, as well as notes from homeopathic courses. For detailed information regarding the availability and the selection process of a homeopathic remedy see Table 1.

**Fig. 2:**
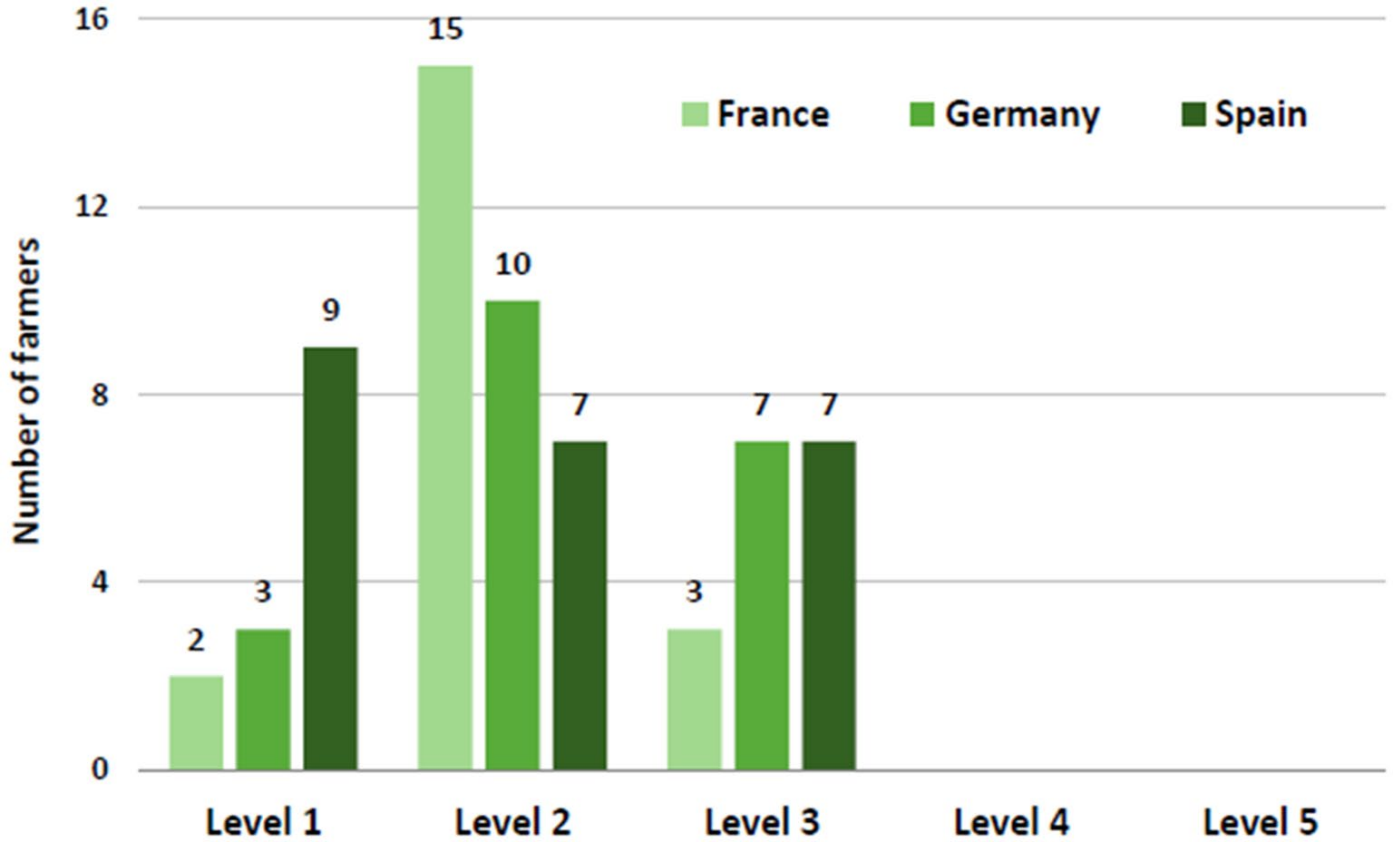
Farmers’ level of awareness of homeopathic principles assessed by IAVH veterinarian

Regarding the competence of farmers in selecting the most appropriate remedy, farmers were rated by the IAVH veterinarians most frequently with level 2 (51%), meaning that they had only basic knowledge in homeopathic principles and often used approved indications. Only a small percentage of the farmers (27%) were capable of administering an individualised homeopathic treatment, and were rated with level 3. A few farmers, rated with level 1 (22%), only used complex remedies or chose a remedy arbitrarily where disease was identified. The top levels, level 4 and 5, were never assigned. Figure 2 shows the assessment results of the farmers’ level of awareness of homeopathic principles. One Spanish farmer was not evaluated as the farmer had never decided which homeopathic remedy to use and consulted the veterinarian in every case.

### Checking treatment outcome

The majority of farmers (83%) stated that they checked the outcome of the treatment (Table 1). However, in most cases, the follow-up checks were only performed visually. Sometimes, the California mastitis test or an udder palpation was carried out. Laboratory investigations were rarely performed. Moreover, farmers were asked if veterinarians regularly checked on the success of their treatments. In total, 70% of all farmers did not consult a veterinarian for the follow-up checks. Assessing the treatment outcome was not (or only in very few cases) performed by local veterinarians in France (0%) and Germany (20%). In Spain, a follow-up check by veterinarians was more common: 63% of farms used this veterinary service and 2 of the Spanish farmers stated that all animals treated were re-checked by a veterinarian. However, the number of animals (all of them or a selection) which were examined by a veterinarian depended on each farmer’s criteria and differed considerably between the countries.

### Documentation

A heterogeneous result was also found in the field of documentation. The majority of farmers did not adequately document their observations and treatments: half of all farmers (50%) documented nothing at all (Table 1). In contrast, 17% of farmers stated that the documentation of treatment (including diagnosis, administration, switching remedies and results of the follow-up check) was always carried out. All other remaining farmers only carried out partial documentation depending on the severity or type of disease, the amount of time available to farmers and the type of treatment. Furthermore, 70% of German, 50% of French and 42% of Spanish farmers did not document homeopathic symptoms. The few remaining farmers took anamnestic records to a varying extent.

## Discussion

The use of homeopathy is controversially discussed in medical science. Although there are many clinical trials concerning the efficacy and/or effectiveness of homeopathic remedies, a clear result as to whether homeopathy is effective or not could not be provided [7, 8]. However, randomised controlled trials focus primarily on the efficacy of the homeopathic remedy itself, whereas the conditions of on-farm use are seldom considered and are rarely a subject of scientific investigations. This study provides a first insight into the existing conditions on dairy farms for the use of homeopathy and on current homeopathic treatment procedures in three European countries. As the number of participants was limited, the representativeness of the study results must be treated carefully. The study results are therefore purely descriptive and do not allow the application of statistical analysis, and more extensive studies are needed in this field.

The most obvious result of the on-farm assessments is the large heterogeneity between farms on how homeopathic remedies are used. The reasons for the heterogeneity in the use of homeopathy are manifold and may include, *inter alia*, the different perspectives and interests of the users, the complexity of the homeopathic treatment approach, and the differences in the availability of homeopathic veterinary remedies or local veterinarians experienced in homeopathy. The study revealed results which were not originally expected. During the inspection of the stall pharmacies, many different homeopathic remedies were found. The main problem here is that approximately three-quarters of these remedies are designed for human use and were not prescribed by veterinarians. Furthermore, colchicine and aristolochia, although prohibited for animal treatment [17], were found in the stall pharmacies. According to EU regulations, only veterinarians are permitted to prescribe human medicinal products for treating food-producing animals [15]. On the other hand, farmers would like to reduce the use of antimicrobial products [18] and are looking for alternatives. The authors are convinced that in the absence of local veterinary advice, farmers find themselves compelled to make decisions on therapy alone or have to resort to pharmacies or non-veterinary practitioners for help. A recently-published study confirmed that the majority of veterinarians had little to no knowledge of the use of alternative therapies, and the majority of veterinarians (72%) were uncomfortable using alternative treatments for livestock [19]. Veterinarians also need to be a minimum familiar with alternative treatment methods in order to be more involved in the treatment process and to discuss the given treatment with farmers. Furthermore, most of pharmacies or non-veterinary practitioners have little or no experience or knowledge of farm animal diseases and are even less well-informed on the legislation covering animal welfare, animal health and public health regulations in livestock production [20]. The study also showed that many famers lack basic training in homeopathy and only had limited knowledge of homeopathic principles. Homeopathy treats each animal as a unique individual, and thus requires individual treatment along with expertise in homeopathic principles. Farmers often hesitate to give an individually tailored treatment and often use *“approved indications”* instead, which contradicts one fundamental principle of individualised homeopathy.

Animal observation was mainly considered insufficient as famers were often distracted by other routine work, resulting in a less thorough detection of diseased animals and the relevant symptoms for homeopathic treatment. A further important finding was the absence of documentation of treatment procedures and outcomes for homeopathic treatment. Farmers might be reluctant to do this because they could be liable to prosecution where using human homeopathic remedies without prescription by a veterinarian when the stall pharmacies are inspected by official veterinarians. Some farmers mentioned the additional work and lack of time as a reason for non-documentation, although farmers are legally obliged to document every treatment given to food-producing animals. Without thorough documentation, a successful outcome cannot be evidenced and farmers cannot learn from treatment failures revealed by monitoring.

The current assessment of the treatment outcome was insufficiently performed by farmers and, in addition, is based on the farmers’ subjective perception. But it is a mistake to think that untreated animals never recover and treated ones always do [21]. Various mastitis studies have shown that untreated animals achieved cure rates of up to 69% [22]. There is no ultimate guarantee for the recovery of udder health where remedies—independent of the therapy method—were administered. For the purpose of evaluating the actual treatment effect, it is therefore absolutely necessary to undertake a clinical examination of each animal being treated. A treatment effect is the difference between the disease outcome with and without treatment [21]. Thorough follow-up checks and documentation of treatment outcomes are required to assess the effects of a change in treatment procedure and to verify the effectiveness of treatments in farm practices [23].

The heterogeneous treatment approaches, together with the often insufficient knowledge of homeopathic principles, do not automatically lead to poor treatment outcomes. A therapeutic success can be achieved in various ways. However, there is an increased risk that factors influencing the outcome of a homeopathic treatment might be overseen or that methodological errors—for example, non-compliance of homeopathic principles during the selection of an appropriate remedy—might occur. The implementation of a *lege artis* treatment procedure can reduce systematic errors, such as an insufficient clinical examination or not checking the treatment outcome. Finally, precise documentation can be expected to lead to the selection of the most appropriate treatment procedures.

However, the actual cure rates of treatment methods are difficult to ascertain at present due to the lack of appropriate follow-up checks of treatment outcomes and documentation. An appropriate treatment monitoring system which enables the assessment of the effectiveness of treatments in farm practices is needed and should be implemented for medical treatments [23]. Many veterinarians hesitate to administer sick animal care using alternative therapies, as their efficacy has not yet been proven. They were concerned that the lack of proven effective therapy options would impair livestock welfare [19]. An appropriate monitoring system could contribute to the assessment of the effectiveness of the homeopathic treatment approach on farms and could satisfy the veterinarians’ need for more data on the efficacy of alternative therapies [19].

After consideration of all the aforementioned facts, the use of homeopathic remedies can currently not be recommended unless a *lege artis* homeopathic treatment procedure and an appropriate initial and boundary conditions on the farm, including the monitoring of treatment outcome, is implemented. These prerequisites are not restricted to homeopathy, but apply also to other alternative treatment methods, especially phytotherapy, and conventional medicine [24]. Without implementing these prerequisites and monitoring systems, it must be assumed that where unsuccessful treatment goes undetected, prolonged suffering of diseased animals will result.

## Conclusion

A target-orientated and successful treatment requires the implementation of a *lege artis* treatment procedure in the use of medicinal products. The study revealed that neither uniform treatment procedures nor a *lege artis* treatment for the use of homeopathy existed on the dairy farms visited. Each farmer seemed to have developed their own homeopathic treatment strategy. This subjective treatment approach using the farmers’ own criteria while neglecting documentation and monitoring is suspected to reduce the potential for successful treatment. The current use of homeopathy carries a high risk for the prolonged suffering of diseased animals in cases where unsuccessful treatment goes undetected. There is, thus, a need to verify the effectiveness of homeopathic treatments in farm practice in consideration of a *lege artis* treatment procedure and homeopathic principles. This can be achieved through regular monitoring of treatment outcomes and the prevalence rate of diseases at herd level. Furthermore, there is a potential risk to food safety due to the use of non-veterinary drugs without veterinary prescription and the use of other prohibited substances in food-producing animals.

## Data Availability

The data set generated and analysed in the current study is not publicly available due to the reason that it contains confidential data which could lead to legal consequences and penalties for farmers. However, the data set is available from the corresponding author on reasonable request.
